# Prevalence of compassion fatigue, burnout, compassion satisfaction, and associated factors among nurses working in cancer treatment centers in Ethiopia, 2020

**DOI:** 10.1186/s12912-023-01383-w

**Published:** 2023-10-10

**Authors:** Almaz Mirutse, Zuriyash Mengistu, Ketema Bizuwork

**Affiliations:** College of Health Sciences, Addis Ababa University, Addis Ababa, Ethiopia

**Keywords:** Compassion Satisfaction, Burnout, Compassion Fatigue, Ethiopia

## Abstract

**Background:**

Overuse of compassion for those under the care may threaten their professional life. In Ethiopia, there is limited study on the consequences of compassionate care. Therefore, the study assessed the effects of compassionate care among nurses.

**Objective:**

To quantify the prevalence of compassion satisfaction, burnout, compassion fatigue, and associated factors among Nurses.

**Methods:**

Institution-based quantitative cross-sectional design was conducted in five randomly selected public hospitals in Ethiopia, from May to April 2020. All the nurses who were working in the cancer treatment centers of the five hospitals were included in the study. Data were collected using a standard self-administer structured question using the Professional Quality of Life Scale (PROQOL) instrument version 5. The data were analyzed by using the SPSS 21version. Descriptively: frequency, mean, standard deviation, and inferential statistics: t-Test and one-way analysis of variance (ANOVA), and multiple linear regression analysis were computed.

**Result:**

The majority of respondents 154 (67.0%) were female. The age of the participants ranges from 20 to 65 (32.06 + 7.45) years. The mean (SD) scores for the dimensions of compassion satisfaction, burnout, and compassion fatigue were 34.41 (6.74), 27.70 (4.24), and 35.83 (7.78) respectively. Neuroticism personality trait had positivity related to compassion fatigue (*P* = 0.001). Nurses who received low monthly income had significantly lower scores for compassion fatigue (*P* = 0.002). We found friend support, openness, sex, and agreeableness explained 32.7% (*p* < 0.024) of the variances in compassion satisfaction.

**Conclusion:**

In general the study found high compassion fatigue and low compassion satisfaction. Further, having low income and neuroticism personality were related to compassion fatigue, while agreeableness, consciousness, and openness personality were related to compassion satisfaction. Therefore, attention should be given to nurses working in cancer centers to ensure positive energy.

## Introduction

Compassionate care for cancer patients may well be a source of both personal fulfillment and intellectual stimulation for healthcare professionals. However, the negative consequences of compassion have been described in terms of compassion fatigue and burnout may threaten professional life in the healthcare system. In particular, professionals working in cancer care are exposed to exact a toll on their physical and emotional health [1]. Compassion fatigue reduced the practitioner’s capacity to be empathic or bear the suffering of clients [2–6]. On the other hand, compassion satisfaction reflects the rewards of caring for others and counterbalances the risks of compassion fatigue [6–13]. The finding of the meta-analysis confirmed that there are levels of risk of suffering from burnout and compassion fatigue among nursing professionals. In that study more women and nurses with more years of experience, with nurses from oncology units were affected, having one of the highest levels of burnout and compassion fatigue [14].

The findings in China showed medium levels of compassion satisfaction, burnout, and compassion fatigue among oncology healthcare professionals, reaching rates of 78.34%, 63.50%, and 75.96%, respectively [15]. According to a meta-analytic estimation, the prevalence revealed 19% for low compassion satisfaction, 56% for medium and high burnout, and 60% for medium and high compassion fatigue [14]. The study in Uganda indicated that close to 50% of the nurses experienced compassion fatigue [6]. A study conducted in Ethiopia showed that 44.4% of nurses had experienced burnout [9].

The factors that contribute to the negative consequence of compassion are demographic data including age, sex, educational status, area of work, years of experience, social support, experience in oncology care areas, position in the work area, and number of patient assignments [4, 6, 14]. Studies revealed older participants presented higher scores of compassion satisfaction, and younger nurses, women nurses having less job experience, and nurses without leisure activities showed higher means of compassion fatigue [6, 8].

In preventing the negative effect of compassionate care, a study identified that nursing staff with self-compassion have a better chance of managing the stresses of their work and care environment [11]. Another study highlighted the importance of recognizing individual signs and symptoms of stress, compassion fatigue, and burnout, and then normalizing stress, compassion fatigue, and burnout for health professionals. Moreover, a study mentioned that professionals have to learn how to manage their stress [16].

Despite there were tried to combat the negative consequences of compassionate care, the problem is not reduced among healthcare providers. Moreover, there is a scarcity of studies in Ethiopia. Therefore, this finding may provide insights for hospital leaders, health programmers, and oncology healthcare providers to develop various strategies to improve the professional quality of life among nursing professionals in oncology centers.

Therefore; this study aims to quantify the prevalence of compassion satisfaction, burnout, compassion fatigue, and associated factors among nurses working in cancer centers of selected hospitals in Ethiopia.

## Methods and materials

### Study setting, study design, and period

The study was conducted in 5 randomly selected public hospitals that provide cancer treatment in Ethiopia. The selected hospitals were Tikur Anbessa Specialized Hospital (TASH), St Paul’s Hospital, Zewditu Hospital in Addis Ababa City, Ayder comprehensive specialized hospital located in Mekelle, Tigray regional State, and Jimma University Medical Center (JUMC) located in Jimma town.

A global cancer observer estimated 77, 352 new cancer cases annually in Ethiopia [17]. There are 44 medical oncologists and 250 nurses in the oncology centers of the country. The lack of capacity of health facilities outside Addis Ababa has resulted in an enormous load of patients from the rest of the country gravitating towards Tikur Anbessa Specialized Hospital in which there are only 19 beds. Tikur Anbessa Specialized Hospital is the only referral oncology center in the country; about 80% of reported cases of cancer are diagnosed at advanced stages [18].

Institutional-based Cross-sectional study design was used from May 2020 to April 2020 in selected hospitals.

### Study participants

The authors considered eight functional oncology centers in Ethiopia including Ayder Comprehensive Specialized Hospital, Gondar Comprehensive Specialized Teaching Hospital, Felege Hiwot Referral Hospital, Hawassa Referral Hospital, Jimma University Medical Center (JUMC), St. Paul’s Hospital, Tikur Anbessa Specialized Hospital and Zewditu Hospital. Among eight hospitals five hospitals were selected using simple random sampling. Tikur Anbessa Specialized Hospital, Ayder Comprehensive Specialized Hospital, St. Paul's Hospital, Jimma University Medical Center. (JUMC). and Zewditu Hospital were the selected areas of the study. Information regarding the study participants was obtained from the human resource management of each institution

The study participants were nurses working in the cancer treatment centers of the selected hospitals excluding those who are critically sick, thus, unable to fill out the questionnaire and have less than six months of working experience in the cancer treatment unit. Hence, few nurses were practiced in the area of the study, all the 250 nurses who work in cancer centers were selected using the census method.

### Operational/terms definition

- *Empathy:* Empathy is the ability to understand and share other people’s emotions and feelings [19, 20].
- *Compassion:* Compassion is a virtuous and intentional response to knowing a person discerns their needs and ameliorates their suffering through relational understanding and action [19].
- *Professional Quality of Life:* Professional quality of life is the internal sense that helpers feel about their work, including both positive and negative aspects [19].
- *Personality traits:* Personality traits are an individual’s behavior toward others that reflect people’s characteristic patterns of thoughts, feelings, attitude, and mindset to make his personality [19].

### Study variables

#### Dependent variable

- Compassion Satisfaction, Compassion Fatigue, and Burnout

#### Independent variables

- Demographic factors are age, gender, marital status, and educational level.
- Work-related factors are work experience, work department, job title, shift, loading, and monthly income in birr.
- Personality traits are extraversion, Agreeableness, Conscientiousness, Neuroticism, and Openness.
- Social factors are social support (significant other support, family support, friend support)

#### Measurement tools

The data was collected using a questionnaire that was developed in the English language and adapted from previously conducted studies in Ethiopia, Nepal, and Spain, [9, 20, 21]. The questionnaire comprised structured questions based on the Professional Quality of Life Scale (PROQOL) instrument version 5. To measure compassion satisfaction for positive feelings and negative feelings of compassion fatigue and burn out we used a self-reported measuring tool. The previously developed ProQOL self-report measuring tool assessed compassion fatigue, compassion satisfaction, and burnout based on how frequently a person has experienced certain antecedents (e.g., “I am happy” or “I feel trapped by my job as a helper”) within the past 30 days. The ProQOL is comprised of 30 items (10 items for each subscale) that are reflective of the three subscales' content. Items are rated on a five-point Likert-type scale, ranging from 1 (never) to 5 (very often). Several items on the Burnout Subscale are reverse-scored [19].

To test the Pro-QOL 5’s reliability within the study sample, Cronbach’s Alpha was computed for each of the measured scales. All three scales indicated a high level of internal consistency. Compassion satisfaction (*α* = 0.831), burnout (*α* = 0.810), and compassion fatigue (*α* = 0.781) had high levels of internal consistency.

The questionnaires focused on the nurses’ level of compassion satisfaction, burnout, and compassion fatigue, and associated factors. The positive and negative ways of compassionate care that can affect the nurse in health care provision were considered [19, 22, 23].

The data collection instruments include four major parts:

- Part I: This section includes questions related to socio-demographic information of the study subjects such as sex, age, educational status, monthly income, and work experience.
- Part II: It includes questions on personality scale questions focused on the individual levels of stress, feeling, and interests.
- Part III: This section includes questions related to the social support of family and friends
- Part IV: The questions ask about the nurse’s work situation that affects them in both positive and negative, as a helping profession.

### Data collection procedure

Approval from the Ethics Committee of the Addis Ababa University, College of Health Sciences, School of Nursing and Midwifery was obtained before data collection. The researchers then contacted the nursing department directors at each hospital and explained the purpose and meaning of this study. Once permission was granted, the head nurses of select departments were invited to serve as research assistants to explain the study’s purpose to the participants. After oral informed consent forms were obtained, the researchers distributed the four-item instruments to the participant. The participants completed the instruments anonymously. All data were numerically coded and accessible only to the researchers to protect confidentiality.

### Data quality assurance

A structured questionnaire was used to collect data after pre-testing the instrument in Armed Force Hospital on 5% of the sample size. The pre-test was conducted two weeks before the actual data collection period and a clear flow in asking the negative and positive questions was maintained. The pretested data were not included in the main study. Complete data collecting instrument preparation takes eight weeks. The data collectors were BSc nurse students who were taken from the same University Hospital in each study area. The training was provided for supervisors and data collectors on the data collection instruments. Then the gap between the method and materials was identified and the appropriate correction was made.

### Data analysis

In this study, data were analyzed by using descriptive statistics including, frequency mean, median, range, standard deviation, and inferential statistics. The normal distribution of the values was tested using the Kolmogorov–Smirnov test. The differences in compassion satisfaction, compassion fatigue, and burnout among participants with demographic and work-related characteristics were tested using the independent t-test and one-way analysis of Variance (ANOVA). Simple regression was performed to assess the relationship between the continuous variables and three dependent ones. Multiple linear regression was computed [20]. Data analysis was done using SPSS 21.0 statistical program.

## Results

### Socio-demographic and work-related characteristics of the respondents

Table 1 shows that out of the total 250 study samples, 230 (92%) individuals responded to our study the majority of respondents accounting for 154 (67.0%) were female. The age of the participants ranged from 20 to 65 (32.06 + 7.45) years. The participants practiced for service age ranged from 1 to 35 (7.75±6.06) years. The oncology unit nursing service experience age ranged from 1 to 16 (4.0 ±2.91) years (Table 1).

**Table 1:** Socio-demographic and work-related characteristics (*N* = 230)

Variables	Category	Number	Percent (%)
Age in years	< 25	36	15.7
	25 – 34	130	56.5
	35 – 44	44	19.1
	> 44	20	8.7
Sex	Male	76	33.0
	Female	154	67.0
Educational Status	Diploma	6	2.6
	Degree	205	89.1
	Master	19	8.3
Marital Status	Single	98	42.6
	Married	120	52.2
	Divorced	11	4.8
	Windowed	1	0.4
Monthly Income in Birr	< 3000	8	3.5
	3000 -5999	97	42.2
	6000- 9999	115	50.0
	> 9999	10	4.3
Area of Work	Pediatric Oncology	75	32.6
	Adult oncology	88	38.3
	^a^another oncology area	67	29.1
Position in Working Area	Head nurse	20	8.7
	Staff Nurse	210	91.3
Work Overload	Yes	180	78.3
	no	50	21.7
Years of Total Clinical Service	< 5	97	42.2
	5– 9	73	31.7
	> 9	60	26.1
Years of Oncology Service	< 5	177	77.0
	5 – 9	41	17.8
	> 9	12	5.2
Duty shift	Day shift	122	53.0
	Night shift	20	8.7
	Alternate shift	88	38.3

*Other oncology area(Hematologic unit, palliative care.)

### Prevalence of compassion satisfaction, burnout, and compassion fatigue

The mean (SD) scores for the dimensions of compassion satisfaction, burnout, and compassion fatigue were 34.41 (6.74), 27.70 (4.24), and 35.83 (7.78) respectively. The median scores (interquartile range) of compassion satisfaction, burnout, and compassion fatigue were 35.00 (30.00– 39.00), 28.00 (25.00–30.00), and 36.00 (31.00–42.00) respectively.

### Univariate analyses of the factors associated with compassion satisfaction, burnout, and compassion fatigue

Table 2 shows the factors associated with compassion satisfaction, burnout, and compassion fatigue. In this study, t-Tests revealed that male nurses had lower compassion satisfaction than female nurses (*p* = 0.007). Nurses who received low monthly income had significantly lower scores for compassion fatigue (*p* = 0.002). Nurses’ educational status was significantly associated with compassion fatigue (*P* = 0.002). Nurses who served a few years in the oncology unit had lower compassion fatigue (*P* = 0.001). Nurses’ duty shift was significantly associated with compassion fatigue (*P* = 0.021).

**Table 2:** Univariate analysis of compassion satisfaction, burnout, and compassion fatigue constructs (*N* = 230)

Variables	Compassion Satisfaction				Burnout			Compassion Fatigue		
	Mean (SD)		t/F	p	Mean (SD)	t/F	p	Mean (SD)	t/F	p
Age in years										
< 25	34.56 (5.74)		2.190	0.090	26.80(4.60)	1.030	0.380	34.94(7.74)	0.198	0.898
25 – 34	34.26(6.98)				27.63(4.11)			35.95(8.06)		
35 – 44	36.07(6.78)				28.25(3.91)			35.98(6.79)		
> 44	31.50(6.07)				28.50(5.06)			36.35(8.54)		
Sex										
Male	32.72(7.88)		2.706	0.007	28.00(4.02)	0.784	0.448	35.38(8.95)	0.613	0.540
Female	35.25(5.96)				27.55(4.35)			36.05(7.16)		
Educational status										
Diploma	35.67(6.56)		0.475	0.622	27.50(2.74)	0.665	0.516	38.83(6.62)	6.185	0.002
Degree	34.50(6.78)				27.80(4.27)			36.27(7.53)		
Master	33.11(6.54)				26.63(4.28)			30.11(8.76)		
Monthly income in birr										
< 3000	26.13(10.34)		4.98	0.002	29.86(5.51)	1.946	0.123	30.63(11.34)	2.256	0.083
3000 -5999	35.34(6.00)				27.10(4.13)			35.01(7.29)		
6000- 9999	34.30(6.61)				28.13(4.24)			36.85(7.64)		
> 9999	33.40(7.95)				26.70(3.47)			36.20(9.53)		
Working area position										
Head nurse		33.40(8.00)	0.633	0.427	28.50(2.72)	1.297	0.205	34.65(8.51)	-0.655	0.520
Staff Nurse		34.51(6.62)			27.62(4.36)			35.94(7.72)		
Work overload										
Yes	34.14(6.86)		-1.171	0.243	27.81(4.50)	0.783	0.435	35.41(7.47)	-1.555	0.121
No	35.40(6.28)				27.28(3.12)			37.34(8.72)		
Years of oncology service										
< 5	34.55(7.21)		0.317	0.729	27.62()	0.210	0.811	34.80(7.70)	7.829	0.001
5– 9	34.22(5.20)				28.07()			38.59(7.28)		
> 9	33.00(3.72)				27.42()			41.58(6.14)		
Duty shift										
Day shift		35.11(6.22)	1.442	0.239	27.52(3.96)	0.916	0.401	36.04(7.51)	3.952	0.021
Night shift		33.95(6.23)			28.90(5.09)			39.90(6.97)		
Alternate shift		33.55(7.48)			27.67(4.42)			34.61(8.06)		

### Simple regression for social support, personality traits factors

Table 3 indicates the linear relationships between the continuous variables and compassion satisfaction, burnout, and compassion fatigue respectively. The personality trait variables in particular agreeableness personality, consciousness, and openness personality had a significant association with compassion satisfaction (all *p* < 0.035). Neuroticism personality trait had positivity associated with compassion fatigue (all *p* = 0.002). Compassion fatigue had significant linear relationships with all of the variables of social support (all *p* < 0.006). In terms of social support variables, friend support had positively related to compassion fatigue (*p* = 0.006). Analyses of the residuals identified that they were normally distributed.

**Table 3:** Simple regression for social support, personality traits, and the Compassion satisfaction, Burnout, and Compassion fatigue constructs (*N* = 230)

Variable	Dimension	Compassion satisfaction			Burnout			Companion fatigue		
		b	SE_b_	p	b	SE_b_	p	b	SE_b_	p
Personality Trait	Extraversion Personality	0.014	0.272	0.841	-0.001	0.185	0.987	0.003	0.318	0.967
	Agreeableness Personality	0.199	0.259	0.008	-0.115	0.176	0.150	-0.083	0.304	0.271
	Consciousness	-0.158	0.244	0.035	0.146	0.166	0.070	-0.101	0.286	0.183
	Neuroticism Personality	-0.078	0.282	0.303	0.082	0.191	0.313	0.238	0.330	0.002
	Openness Personality	0.336	.346	0.000	-0.154	0.235	0,071	-0.105	0.405	0.188
Social support	Family Support	0.118	0.362	0.100	-0.155	0.254	0.054	-0.274	0.456	0.001
	Significant Others Support	.0.094	0.293	0.142	-0.015	0.206	0.837	-0.202	0.369	0.004
	Friend Support	0.377	0.429	0.000	-0.119	0.301	0.132	0.214	0.539	0.006

### Multiple linear regressions statistical analysis of predictors

According to Table 4, the results of multiple linear regression analysis demonstrated that four variables of friend support, openness, sex, and agreeableness explained 32.7% (*p* < 0.024) of the variance in compassion satisfaction. Family support accounted for 4.7% (*p* < 0.001) variables in burnout. Furthermore, six variables of neuroticism, oncology service, educational status, family support, friend support, and significant other support explained 26.3% (*p* < 0.04) of the variance in compassion fatigue.

**Table 4:** Multiple linear regression statistical analysis of compassion satisfaction, burnout, and Compassion fatigue among oncology nurses in selected Ethiopian hospitals, (*N* = 230)

Model	b	SE_b_	b’	t	p	R^2^ change	F	p	R-square	Adjusted R^2^
Compassion satisfaction										
Constant Term	10.329	2.421		4.267	0.000		5.173	0.024	0.339	0.327
Friend Support	2.297	0.344	0.378	6.675	0.000	0.226				
Openness	1.044	0.268	0.237	3.900	0.000	0.077				
Sex	2.326	0.788	0.163	2.953	0.003	0.021				
Agreeableness	0.478	0.210	0.136	2.274	0.024	.015				
Burnout										
Constant Term	31.220	1.044		29.91	0.000		12.247	0.001	0.051	0.047
Family Support	-0.721	0.206	0.226	-3.500	0.001	0.051				
Compassion Fatigue										
Constant term	39.839	4.153		9.592	0.000		4.279	0.040	0.282	0.263
Neuroticism	1.070	0.257	0.248	4.165	0.000	0.128				
Oncology Service	0.673	0.158	0.251	4.272	0.000	0.056				
Educational Status	-5.211	1.384	0.218	-3.766	0.000	0.049				
Family Support	-1.157	0.417	0.198	-2.775	0.006	0.18				
Friend Support	1.294	0.489	0.185	2.644	0.009	0.17				
Significant Other Support	-0.700	0.338	0.131	-2.068	0.040	0.14				

The assumptions of normality, independence, linearity, and homoscedasticity for multiple linear regressions were checked.

## Discussion

This study was conducted to measure levels of compassion satisfaction, burnout, and compassion fatigue among nurses who work in oncology care units by using the Professional Quality of Life Scale (ProQOL R-V) at five hospitals in Ethiopia [19, 23]. Registered nurses (*N*=230) from oncology care units completed the demographic and Professional Quality of Life Scale, Version 5 (ProQOL5) questionnaire. In this study, the greatest number of the study participants had a BSc degree (89.1%) educational level. More than half of the respondents were female (67.0%). The study revealed that the mean score of compassion satisfaction, burnout, and compassion fatigue were 34.41, 27.70, and 35.83 respectively. The compassion satisfaction and burnout result is in agreement with the study conducted in Kenya which had a 38.47 mean score of compassion satisfaction and 24.20 burnout, and compassion fatigue in the study (26.33) [4].

This study revealed that male nurses had lower compassion satisfaction than female nurses (*p* = 0.007). This finding is consistent with the results of another study conducted in Portugal [8]. It suggests that males in our setting hardly manage their stress. Friend support, openness, sex, and agreeableness explained 32.7% of the variance in compassion satisfaction. The possible explanation might be attributable to an increased personal stress-managing ability and a good social support network, Thus, good social support could alleviate the stressful effects of patient care and serve against burnout and compassion fatigue [12].

We found that the personality trait variables in particular agreeableness, consciousness, and openness personality contributed to compassion satisfaction (*p* < 0.035). This finding is consistent with the study conducted in China [23]. It suggests that open nurses may engage in more activities that enhance their satisfaction with caring activities. Similarly, conscientious nurses are prudent, and hardworking and set high standards for themselves.

In this study, family support accounted for 4.7% of the variables in burnout. This finding is in agreement with the study conducted in China that indicates social support acted as a protective predictor of burnout [23].

The six variables of neuroticism, oncology service, educational status, family support, friend support, and significant other support explained 26.3% of the variance in compassion fatigue. This finding is in agreement with a study conducted in China [23]. The possible expansion could be neurotic nurses may have a greater inability to control their emotions when faced with negative events, putting them at higher risks of compassion fatigue.

### Limitations of the study

The limitation of the study includes: first, it could be noted from the finding of this study was a small sample size due to the lower number of oncology nurses to draw a random sample. Second, the study also used self-report instruments with the possibility of recall bias because the reliability of collected data can be affected by the respondents’ interests and attitudes. Third, this study used a single tool that did not measure the quality of nurses’ profession comprehensively. Finally, the study also measured compassion satisfaction; burnout, and compassion fatigue at a single point in time, and the responses could change over time depending on respondents’ personal and employment situations.

## Conclusion

In general the study finds out that oncology nurses have high compassion fatigue and low compassion satisfaction. Compassionately assisting patients over a long period makes oncology nurses prone to suffer from compassion fatigue. This finding suggests that nurses may be lacking sufficient skills in coping with the traumatic experiences of their patients. Being male, having low income, having longer work experience, and neuroticism personality were related to compassion fatigue, while agreeableness, consciousness, and openness personality were related to compassion satisfaction. It could be concluded that oncology nurses in Ethiopia are under a great deal of hassle and work burdens which leave them defenseless against the winds of Compassion Fatigue and Burnout Out with less Compassion Satisfaction. Therefore, attention should be given to oncology nurses to ensure positive energy, emotional intelligence, and emotional strength.

## Recommendations

This result can be used by nursing leadership and other stakeholders to create an enabling environment for nurses to promote compassion satisfaction and avoid Compassion Fatigue and Burnout in the nursing workforce.

Nursing educators should promote relevant on-duty training for oncology nurses and raise their awareness of both the possible negative influences of working with cancer patients and the potential for compassion satisfaction.

Further studies should be conducted in different specialties nurses to promote the generalizability of findings and explore other potential predictors.

## Data Availability

All relevant data are included within the manuscript document. If it is necessary, it is possible to contact the corresponding author to get additional materials.

## Abbreviations

AAU: Addis Ababa University
CHS: College of Health Science
BO: Burn Out
CF: Compassion Fatigue
ProQOL: Professional Quality of Life Scale
